# Ultrasound-assisted binary probiotic fermentation of sugarcane bagasse dietary fiber-containing synbiotics yogurt: Increased galactitol content to improve yogurt rheological properties and flavor^☆^

**DOI:** 10.1016/j.ultsonch.2026.108118

**Published:** 2026-10-15

**Authors:** Hui Zhou, Wenying Yuan, Chengfeng Xu, Xiefei Li, Zhen Wu, Daodong Pan, Jie Luo, Xiankang Fan

**Affiliations:** a School of Public Health, The Key Laboratory of Environmental Pollution Monitoring and Disease Control, Ministry of Education, Guizhou Provincial Engineering Research Center of Ecological Food Innovation, Guizhou Medical University, Guiyang 561113, China; b College of Food Science and Technology, Hunan Agricultural University, Changsha 410128, China; c Key Laboratory of Animal Protein Food Processing Technology of Zhejiang Province, College of Food and Pharmaceutical Sciences, Ningbo University, Ningbo 315832, China

**Keywords:** Lactic acid bacteria, Metabolomics, Electronic nose, HS-SPME-GC–MS, UHPLC-MS

## Abstract

The effect of ultrasound-assisted binary probiotic fermentation on the quality of sugarcane bagasse dietary fiber (SBDF)-added synbiotics yogurt were investigated. The results showed that 0.25 % (w/v) of SBDF significantly reduced yogurt syneresis. Furthermore, ultrasound-assisted fermentation with *L. plantarum* CGMCC 1.5953 and *L. fermentum* CGMCC 1.7434 improved the yogurt’s texture, rheology, microstructure, and sensory scores. These improvements were supported by metabolomic analysis, which showed an 11.91-fold upregulation of galactitol and decreased concentrations of L-phenylalanine, L-glutamic acid, and L-allothreonine. Integrated sensory and molecular analyses (HS-SPME-GC–MS, UHPLC-MS) identified that enhanced texture scores were associated with SBDF-derived metabolites, including L-erythrulose, α-lactose, and erythritol. Meanwhile, the improved odor scores were associated with increased levels of ethyl acetate, hexanoic acid, and 2-nonanone. Key pathways involve galactose, tyrosine, glycine-serine-threonine metabolism, amino acid biosynthesis, and lysine degradation. This study provides valuable insights for the high-value utilization of sugarcane bagasse dietary fiber and the production of premium-quality yogurt.

## Highlights

• Dietary fiber-rich and unique flavor of synbiotics yogurt.
• Ultrasound-assisted fermentation improves yogurt flavor and rheological properties.
• Increased levels of metabolized galactitol in LAB under ultrasonic stress is critical.
• Ethyl acetate, hexanoic acid, and 2-nonanone was attributed to odor improvement.
• Mainly concerned with galactose metabolism and amino acid metabolism.

## Graphical abstract

**Figure:**
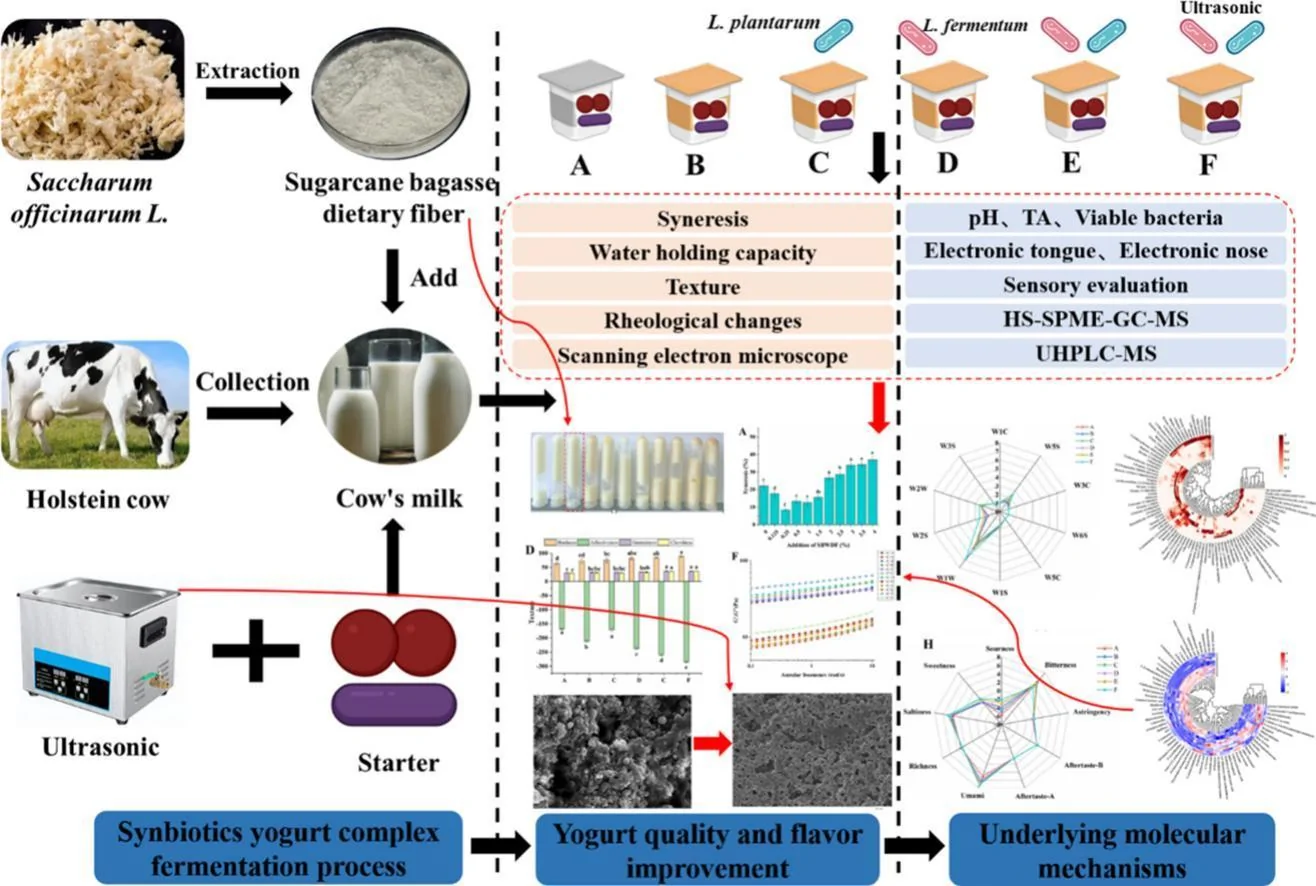

## 1 Introduction

Yogurt is made from cow’s milk, pasteurized and fermented with starters (*Streptococcus thermophilus* and *Lactobacillus delbrueckii* subsp. *bulgaricus*). It is popular among consumers because of its unique flavor (mellow, moderately sweet and sour, etc.) and probiotic functions (regulating intestinal flora, alleviating lactose intolerance, lowering cholesterol, etc.) [1]. It is also considered an effective carrier of nutrients such as probiotics, prebiotics, proteins, vitamins, and minerals. For example, Zhu et al. found that probiotic yogurt significantly reversed body and liver weight gain[2]. It also reversed hepatic steatosis and liver damage caused by a high-fat diet. The relative abundance of short-chain fatty acid-producing bacteria was increased. This increase was achieved by decreasing the proportion of the phylum Firmicutes/Bacteroidota. However, plain yogurt has problems such as easy whey precipitation, poor gel strength, and single flavor [3]. Therefore, the use of non-dairy milk (plant milk) or the production of yogurt or the addition of natural plant-based extracts not only improves the chemical composition of yogurt and confers additional probiotic functions, but also improves the microstructure, color, texture, and flavor of yogurt. These additions act as natural stabilisers, colouring agents and flavour enhancers [4]. For instance, Lin et al. found that the incorporation of ginkgo polysaccharides enhanced the accumulation of organic acids, water-holding capacity, and antioxidant activity in yogurt[5]. These improvements were achieved by promoting the formation of protein and oil-body clusters. This led to the formation of a more compact gel network in the yogurt. Furthermore, Sharifi et al. reported that the use of carrot pomace extract significantly increased the survival of probiotics in yogurt during storage [6]. In addition, consumer demand for healthier features, more natural ingredients and newer flavors is gradually increasing [7]. Therefore, the development of novel yogurts with uniform texture, unique flavor and probiotic potential is of strategic research importance.

Dietary fiber (classified as soluble and insoluble) is derived from plant material and consists of complex non-starchy carbohydrates and lignin. It is indigestible in the small intestine, as the necessary enzymes are not produced by mammals; these enzymes would hydrolyse the polymers into their constituent monomers [8]. They can reach the colon intact and are fermented by the gut microbiota [9]. For example, in the exploration of soluble dietary fibre fermentation by gut microbiota, it was found by Bai et al. that the biosynthetic pathway of short-chain fatty acids was related to the chain structure of soluble dietary fibre polysaccharides [10]. The enrichment of *Lactobacillus* spp. was promoted by the soluble dietary fibre. Notably, Yang et al. found that dietary fiber and yogurt intake were associated with a reduced risk of lung cancer in never-smokers in a two-year survey of 1,445,850 adults from the US, Europe and Asia[11]. Nevertheless, Tomic et al. showed through cluster analysis that black wheat insoluble dietary fiber fortified the texture of yogurt, but consumer acceptance and quality scores were low [12]. Therefore, soluble dietary fiber may have a greater opportunity to improve the texture or flavor of yogurt compared to insoluble dietary fiber [13]. The soluble dietary fiber content of sugarcane has been reported to be 20 %, which is higher than that of soya beans, maize and wheat. It is free of phytic acid and is primarily found in bagasse, a by-product of processing that is inexpensive [14]. However, the processing method suitable for the utilization of bagasse dietary fiber in yogurt is unclear.

Additional lactic acid bacteria (LAB) are usually added to enhance the special probiotic properties of yogurt and to improve its flavor or texture characteristics. Probiotic strain selection for yogurt prioritizes acid/bile tolerance, storage viability, sensory contribution, and health benefits. Key fermentation traits include acidification rate, exopolysaccharide production, and flavor/bioactive metabolite generation. For example, Fan et al. found that yogurt fermented by *Levilactobacillus brevis* CGMCC 1. 5954, a highly GABA-producing bacterium, was able to alleviate circadian rhythm disorders in mice via the gut-brain axis[15]. The addition of *Lactobacillus paracasei* ZFM54 to fermented yogurt not only improves acidity, water retention and the number of live LAB, but also increases the volatile acid content and improves the flavor [16]. However, Peng et al. demonstrated that binary probiotics (*Lacticaseibacillus casei* Zhang and *Bifidobacterium lactis* V9) may be better equipped to withstand adverse environments compared to single strains, and significantly higher levels of acetic, caproic, butyric, and valeric acids were observed in yoghurts fermented with binary probiotics during storage [17]. Higher levels of flavor compounds acetaldehyde, diacetyl, ethyl diphosgene and acetic acid were found in yogurt fermented with the combination of *Lacticaseibacillus* Zhang and *Bifidobacterium animalis* ssp. *lactis* Probio-M8 [18]. Nevertheless, further examples and context-specific analyses are needed to substantiate the role of binary probiotics. In this context, strain selection is based on complementary functional traits. In this context, specific strains are selected based on their complementary functional traits. Separately, a strain of *Lactiplantibacillus plantarum* subsp. *plantarum* CGMCC 1.5953, highly efficient in degrading cholesterol, and *Limosilactobacillus fermentum* CGMCC 1.7434, capable of producing high levels of antioxidant peptides, were pre-screened [19], [20]. Therefore, we speculate that it is feasible to use them to synergistically ferment synbiotic yogurt with prebiotic potential from bagasse dietary fiber and to elucidate the mechanism of their interspecies interactions on yogurt quality.

In this study, the effect of binary probiotics synergizing with sugarcane bagasse dietary fiber (SBDF) on yogurt quality under ultrasonic pre-treatment and its molecular mechanisms were investigated. First, water-soluble dietary fiber was extracted from bagasse and screened for optimal addition to yogurt based on syneresis rate. From a broader perspective, the use of sugarcane bagasse, an agricultural by‑product, aligns with sustainable food processing practices. It converts waste streams into valuable functional ingredients. Secondly, the effects of ultrasonic pretreatment, bagasse dietary fiber and binary probiotics on yogurt pH, acidity, water holding capacity, texture, rheology and microstructure were investigated. Finally, the effects on yogurt flavor and their molecular mechanisms were investigated by electronic nose, electronic tongue, sensory evaluation, Headspace-Solid Phase Microextraction-Gas Chromatography-Mass Spectrometry‌ (HS-SPME-GC–MS), and Ultra-High Performance Liquid Chromatography-Mass Spectrometry‌ (UHPLC-MS) combined with molecular sensory science. It is important to note that the present work focuses on the mechanistic interplay among soluble dietary fiber, binary probiotics, and ultrasound treatment in shaping yogurt quality, rather than on the optimization of fiber extraction technology per se. The extraction procedure was employed as a means to obtain a compositionally stable SBDF material suitable for controlled mechanistic studies. This study is essential for the development of novel synthetic metabolic yogurt, high quality utilization of bagasse and elucidation of the mechanism of synergistic fermentation of yogurt by binary probiotics under ultrasonic pre-treatment.

## 2 Materials and methods

### 2.1 Materials

*Limosilactobacillus fermentum* CGMCC 1.7434 and *Lactiplantibacillus plantarum* subsp. *plantarum* CGMCC 1.5953 were screened by Ningbo University and deposited in CGMCC, and the starters consisted of *Streptococcus thermophilus* CGMCC 1.1864 and *Lactobacillus delbrueckii* subsp. *bulgaricus* CICC 6045 were preserved in Hunan Agricultural University. The sugarcane is white jade sugarcane grown in Guangxi, China, and the whole milk powder and sucrose are produced by Anchor (Auckland, New Zealand) and Anqi Yeast Co Ltd (Huibei, China). Methanol (≥99.0 %) and acetonitrile (≥99.0 %) are produced by Thermo (Massachusetts, USA), while 2-chloro-L-phenylalanine (internal standard reference material, 98 %), formic acid (LC-MS grade) and ammonium formate (≥99.9 %) are provided by Aladdin (Washington, USA), TCI (Shanghai, China) and Sigma (Shanghai, China) respectively.

### 2.2 Extraction of dietary fiber from sugarcane bagasse

This conventional alkaline-enzymatic extraction method was employed to obtain a well-characterized SBDF material suitable for mechanistic investigations. Firstly, the impurities were removed from the juiced bagasse, which was coarsely crushed using an ultra-micron crusher (Zhejiang Deqing Baijie Electric Co., Ltd., Zhejiang, China), and then sieved through a 200-mesh sieve. Water was added according to 1:5 (w/v) and steamed at 100 °C for 4 h. After cooling to 65 °C (the optimal temperature for enzyme activity), 0.3 % (w/v) of α-amylase and protease were added (the mass ratio of α-amylase to protease was 1:3) and the mixture was incubated at this temperature for 90 min to ensure effective enzymatic hydrolysis. The enzymes were then inactivated at 100 °C. Subsequently, a mixed solution of 2.5 % NaOH and 17 % (w/w) anhydrous sodium sulfite was added, and the reaction was carried out under magnetic stirring, followed by filtration. The filtrate was soaked and washed with distilled water five times, then concentrated and dried using a spray dryer [21]. The resulting bagasse was subjected to the same washing and drying procedure. After being sieved again, the bagasse was sterilized at 121℃ for 15 min, and the sterilized sugarcane bagasse dietary fiber (SBDF) was collected and set aside [22].

### 2.3 Effect of different SBDF additions on yogurt syneresis

According to the method of Fan et al. with slight modifications, explore the influence of different SBDF contents on yogurt syneresis was explored [23]. Reconstituted milk containing 5 % (w/v) whole milk powder, 6.5 % (w/v) sucrose, and different concentrations of SBDF (0, 0.125 %, 0.25 %, 0.5 %, 1 %, 1.5 %, 2 %, 2.5 %, 3 %, 3.5 %, and 4 % (w/v)) was homogenized at 65 °C and 17 MPa for 25 s, heat-treated at 95 °C for 10 min, and then cooled to 42 °C. SBDF was added at concentrations of 0, 0.125 %, 0.25 %, 0.5 %, 1 %, 1.5 %, 2 %, 2.5 %, 3 %, 3.5 % and 4 % (w/v) respectively. Inoculate the starter (*S. thermophilus* and *L. bulgaricus*) at 6.5 × 10^6^ CFU/mL, and then place it at 42 °C for fermentation for 6 h. After fermentation, the yogurt was stored at 4 °C for 12 h for post-ripening. Syneresis was measured according to the method described by Wang, Kristo, & LaPointe [24]. Accurately weigh 20 g of the fermented yogurt, centrifuge it at 10℃ and 222 × g for 10 min, aspirate the supernatant and weigh its weight, and calculate the syneresis of the yogurt according to Formula 1.

(1) $$\mathrm{Syneresis}\left(\%\right)=m_{2}/m_{1}\times 100$$

Here, *m_1_* stands for the weight of the original yogurt sample, and *m_2_* stands for the weight of the obtained yogurt supernatant.

### 2.4 Preparation of yogurts containing SBDF

Based on the syneresis results, an SBDF concentration of 0.25 % (w/v) was selected for subsequent yogurt production. A base mixture containing 0.25 % SBDF, cow milk, and 6.5 % (w/v) sucrose was homogenised (65 °C, 17 MPa, 25 s), heat-treated, and cooled to 42 °C. The yogurts were divided into six groups: group A was only inoculated with the starter; group B was added with 0.25 % (w/v) SBDF and inoculated with the starter; group C was added with 0.25 % (w/v) SBDF, the starter and *L. plantarum* CGMCC 1.5953; group D was added with 0.25 % (w/v) SBDF, the starter and *L. fermentum* CGMCC 1.7434; group E was added with 0.25 % (w/v) SBDF, the starter, *L. plantarum* CGMCC 1.5953 and *L. fermentum* CGMCC 1.7434; group F was added with 0.25 % (w/v) SBDF, the starter, *L. plantarum* CGMCC 1.5953 and *L. fermentum* CGMCC 1.7434 and was ultrasonically treated at 100 W for 30 min based on previous optimization study on the ultrasonic treatment of lactic acid bacteria [25]. All groups were inoculated with a total of 6 × 10⁶ CFU/mL of the respective lactic acid bacteria, fermented at 42 °C for 6 h, and subsequently stored at 4 °C for 12 h for post-ripening.

### 2.5 Effect of ultrasound, binary probiotic fermentation, and SBDF on the number of viable bacteria, pH, and titratable acidity (TA) of yogurt

Determine the viable count in yogurt with slight modifications according to the methods used by Peng et al. [17] and Fan et al. [26]. The content of *S. thermophilus* in yogurt was obtained by diluting it 10^9^-fold with sterile normal saline, inoculating it onto M17 medium, and counting after culturing at 37 °C for 24 h. The content of *L. bulgaricus* was determined after culturing on MRS medium under anaerobic conditions at 37 °C for 48 h. *L. fermentum* CGMCC 1.7434 was counted after culturing on MRS medium in an anaerobic incubator at 36 ± 1 °C for 7 ± 2h. *L. plantarum* CGMCC 1.5953 was cultured in sorbitol medium (in which the glucose in MRS medium was replaced by an equal amount of sorbitol) in an anaerobic incubator at 37℃ for 48 h. Measure the pH value of the fermented yogurt using a pH meter (Sartorius PB-10, Germany). Determine the total acidity of the yogurt with slight modifications according to the method of Shi et al. [27]. Measure out 25 mL of the yogurt sample, add 40 mL of water and 0.2 mL of 1 % phenolphthalein indicator solution, and then titrate with 0.1 mol/L sodium hydroxide solution. TA was calculated as:

(2) $$\mathrm{TA}\left({\;}^{{}^{\circ}}T\right)=\frac{V\times N\times k\times 100}{W}$$

where *N* is the normality of NaOH, *k* is the milliequivalent weight of lactic acid (0.009 g/meq), and *W* is the sample weight (g).

### 2.6 Effects on water holding capacity, texture, and rheological properties of yogurt

Following the method of Lee & Lucey [28], with minor adjustments, the texture of yogurt was measured using a texture analyzer (Stable Micro Systems, UK). A probe with a diameter of 12.7 mm was employed. The pre-test and test speeds were both set at 1.0 mm/s, and the post-test speed was set at 2.0 mm/s. The water-holding capacity of the fermented yogurt was measured with slight modifications as per the method reported by Wang et al. [29]. Weigh 20 g of yogurt and centrifuge it at 1,000 × g and 4 °C for 10 min. WHC was quantified using the equation:

(3) $$\mathrm{WHC}\left(\%\right)=\frac{{(W}_{0}-W_{1})\times 100}{W_{2}}$$

Here, *W_0_* stands for total weight of centrifuge tube plus yogurt before centrifugation, *W_1_* stands for total weight of tube plus pellet after supernatant removal, and *W_2_* stands for initial yogurt weight.

As reported by Ren et al. [30], the rheological properties of yogurt were measured using a DHR-2 rheometer (TA-WATERS, USA). The fixed frequency was 1 Hz, the strain range was from 0 to 50 %, the shear rate was increased from 0 to 500 s^−1^ and then decreased from 500 to 0 s^−1^, covering a frequency range of 0.1–10 Hz.

### 2.7 Microstructure of yogurt under scanning electron microscope

First, take a fermented yogurt sample with a size of approximately 1.5 × 1.5 × 1.5 cm. Fix it in 2.5 % glutaraldehyde at 4 °C for 12 h, and then rinse it aseptically twice. Then, perform gradient dehydration on it using ethanol (50 %, 60 %, 70 %, 80 %, and 90 %), with each treatment lasting for 10 min [23]. Finally, wash it with 100 % ethanol three times. After being immersed in *tert*-butanol for 10 min, pre-cool it at −80 °C for 24 h, and then freeze-dry it in a freeze-dryer for 12 h. After gold − sputtering treatment, observe the sample at 10,000 × magnification using a ZEISS Sigma 500 scanning electron microscope (Zeiss, Baden − Württemberg, Germany).

### 2.8 Impact on sensory evaluation and electronic tongue

Sensory evaluation was conducted by 26 trained volunteers (13 males and 13 females who engage in food-related professions, average aged 24-year-old) on the color (20 points), texture (30 points), odor (20 points) and taste (30 points) of six types of yogurts at room temperature [31]. This was approved by the Ningbo University Institutional Review Board (NBU20220189). All volunteers were informed that the test products were safe for consumption. They had signed the informed consent form and could withdraw from the experiment at any time without giving reasons. Sensory scores were expressed as mean ± SD (n = 26), and differences among groups were analyzed by one-way ANOVA with Duncan's test (*P* < 0.05). Take a yogurt sample and mix it with an equal volume of deionized water at a ratio of 1:1. Then load it into a 50 mL centrifuge tube. Centrifuge the tube containing the sample at 3000 × g for 5 min. After centrifugation, draw the supernatant and use the TS-5000Z electronic tongue (INSENT, Japan) for analysis. Use a 0.3 mM tartaric acid and 30 mM potassium chloride solution as the reference [32]. Electronic tongue data were processed using the instrument's built-in software, and sensor response values were normalized to the reference solution.

### 2.9 Electronic nose analysis

Following the method of Zhao et al. [33] with minor changes, the PEN3 electronic nose (AIRSENSE, Mecklenburg-Vorpommern, Germany) was employed to identify the volatile flavor profile of yogurt. The sensors were W1C, W1S, W1W, W2S, W2W, W3C, W3S, W5S, W5C, and W6S. Measure 20 mL of yogurt and put it into a 50 mL centrifuge tube. Then, seal it with plastic wrap and leave it at 26 °C for 30 min. Use an extraction needle to prick the plastic wrap on the centrifuge tube. After each test, completely flush the sensors with fresh air for 90 s to get rid of the remaining odor molecules. Sensor response values were normalized to the baseline (clean air). A response intensity threshold of ≥ 1.5-fold over baseline was considered significant for sensor signal interpretation.

### 2.10 Determination of volatile flavor compounds in yogurt based on HS-SPME-GC–MS

According to the methods used by Dan et al. [34] and Fan et al. [35], with slight modifications, the volatile flavor substances in yogurt were determined. Weigh 5 g of yogurt into an extraction flask, and extract the volatile flavor compounds at 350 × g and 55 °C for 1 h. For the Agilent 8890 GC System + 5977B/MSD (Agilent, California, USA), the carrier gas is He with a flow rate of 1.0 mL/min, the inlet temperature is set at 250 °C. The temperature-ramping program starts at 35 °C and is maintained for 5 min, then gradually increases to 140 °C at a rate of 5 °C/min and is maintained for 2 min. Subsequently, the temperature is increased to 250 °C at a rate of 10 °C/min and is maintained for 3 min.

### 2.11 Determination of non-volatile flavor compounds in yogurt based on UHPLC-MS whole-target metabolomics

Transfer 1 mL of yogurt into a 2 mL centrifuge tube, add 400 µL of methanol solution and vortex for 1 min. Then centrifuge at 12,000 × g for 10 min at 4 °C. Take the supernatant and concentrate it to dryness. Add 150 µL of 2-chloro-L-phenylalanine (4 ppm) solution prepared with 80 % methanol–water to re-dissolve the above yogurt sample. After passing through a 0.22 μm membrane for filtration, it is used for UHPLC- MS detection [36]. The Thermo Vanquish ultra-high-performance liquid chromatography system (Thermo Fisher Scientific, USA) was employed, along with an ACQUITY UPLC® HSS T3 column (2.1 × 100 mm, 1.8 µm, Waters, Milford, MA, USA). The flow rate was set at 0.3 mL/min, the column temperature at 40 °C, and the injection volume at 2 μL. Data collection was carried out using the Thermo Q Exactive Focus mass spectrometry detector (Thermo Fisher Scientific, USA). The positive-ion spray voltage was 3.50 kV, while the negative-ion spray voltage was −2.50 kV. High-energy collisional dissociation was utilized for second-level fragmentation, with a collision energy of 30 eV and a second-level resolution of 17,500. For differential volatile compound screening, VIP ≥ 1.0, fold-change ≥ 1.5 or ≤ 0.667, and *p* < 0.05 were applied as cut-off criteria.

### 2.12 Statistical analysis

All experiments were performed in at least three independent replicates and presented as mean ± SD. Statistical significance (*P* < 0.05) was determined by one-way analysis of variance (ANOVA) followed by Duncan’s multiple range test using SPSS 26.0. The Simca software and the website (https://www.bioladder.cn/web/#/pro/index) were used to draw the principal coordinate analysis (PCoA) diagrams and heat maps. The Kyoto Encyclopedia of Genes and Genomes (KEGG) was used for metabolic pathway analysis, while the websites (https://flavornet.org/flavornet.html) and (https://mffi.sjtu.edu.cn/) were used to annotate the aroma profiles of flavor compounds.

## 3 Results and discussion

### 3.1 Effect of SBDF on yogurt syneresis

Plain yogurt production encounters several technical difficulties, such as poor gel formation, limited flavour variety, and whey separation [3]. This latter phenomenon, known as syneresis, greatly reduces product quality and consumer appeal. It is often visible as whey pooling on the surface of the yoghurt [37]. As shown in Fig. 1, the color of the yogurt gradually turned yellow as the concentration of SBDF increased. No sedimentation of SBDF was observed in any of the fermented samples. However, after centrifugation and inversion of the yogurt, whey precipitation and breakage occurred in all groups except for the addition of 0.25 % (w/v) SBDF. This is mainly due to the fact that the extracted SBDF is divided into soluble and insoluble dietary fibers, and centrifugation disrupts the three-dimensional gel network structure of the yogurt, causing the insoluble dietary fibers to precipitate and settle to the bottom [38]. The yogurt syneresis was significantly reduced to 8.42 % at the SBDF addition of 0.25 % (w/v) compared to the group without added SBDF (Fig. 2-A). It was shown that both coarse and fine fibers accelerate structure formation during yogurt fermentation. In particular, yoghurt supplemented with low concentrations of orange fibre exhibited reduced syneresis [39]. This suggests that the extracted SBDF contains a higher proportion of fine fibres. These may be favoured by the presence of pectin, which is released from SBDF during mechanical and thermal pre-treatment. Due to its calcium sensitivity, this pectin may help bridge casein micelles and stabilise the gel structure.

**Fig. 1:**
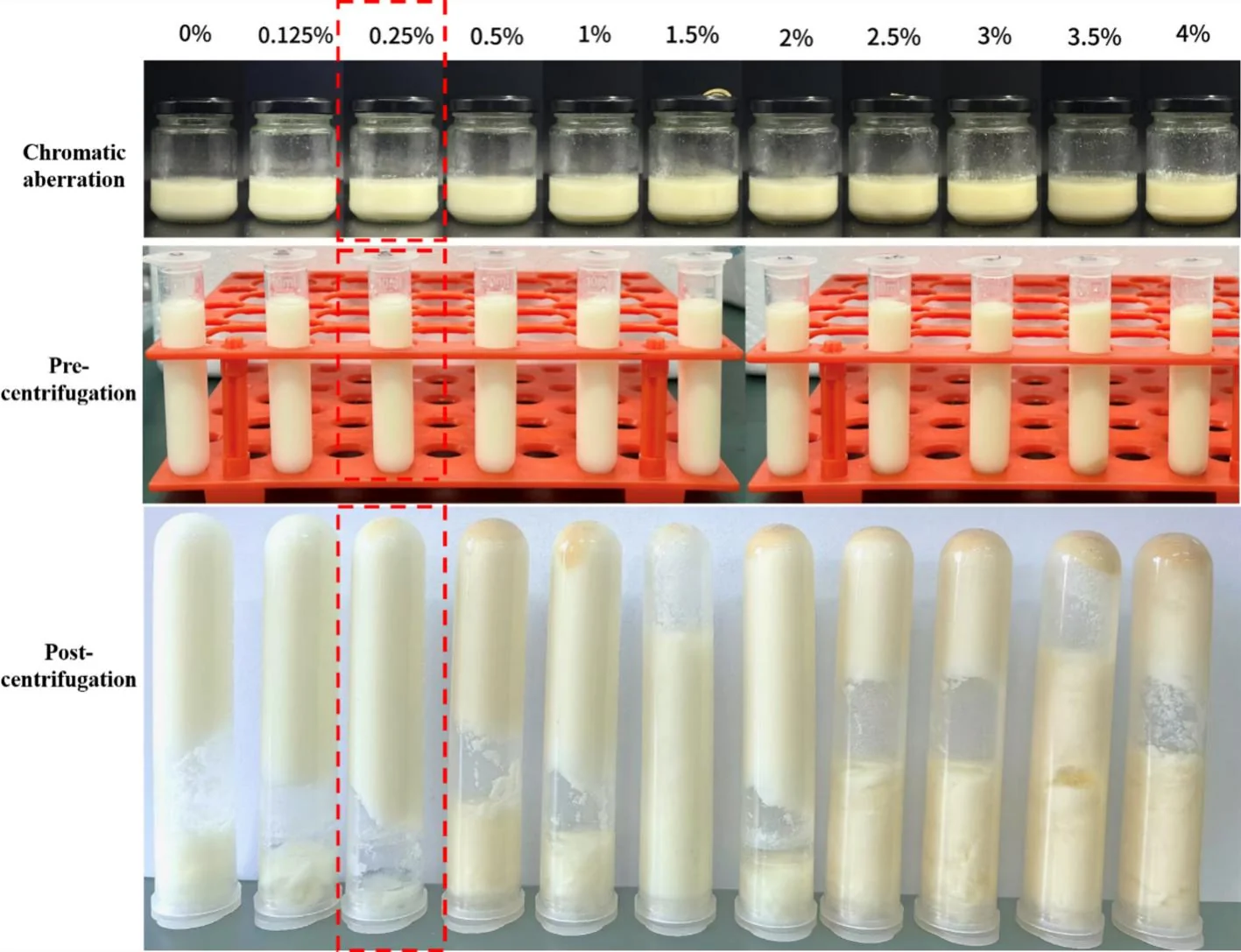
Effect of different concentrations of SBDF on the apparent appearance of yogurt. (A): Effect on apparent color. (B): Pre-centrifugal tissue state. (C): Centrifuge and invert the tissue state.

**Fig. 2:**
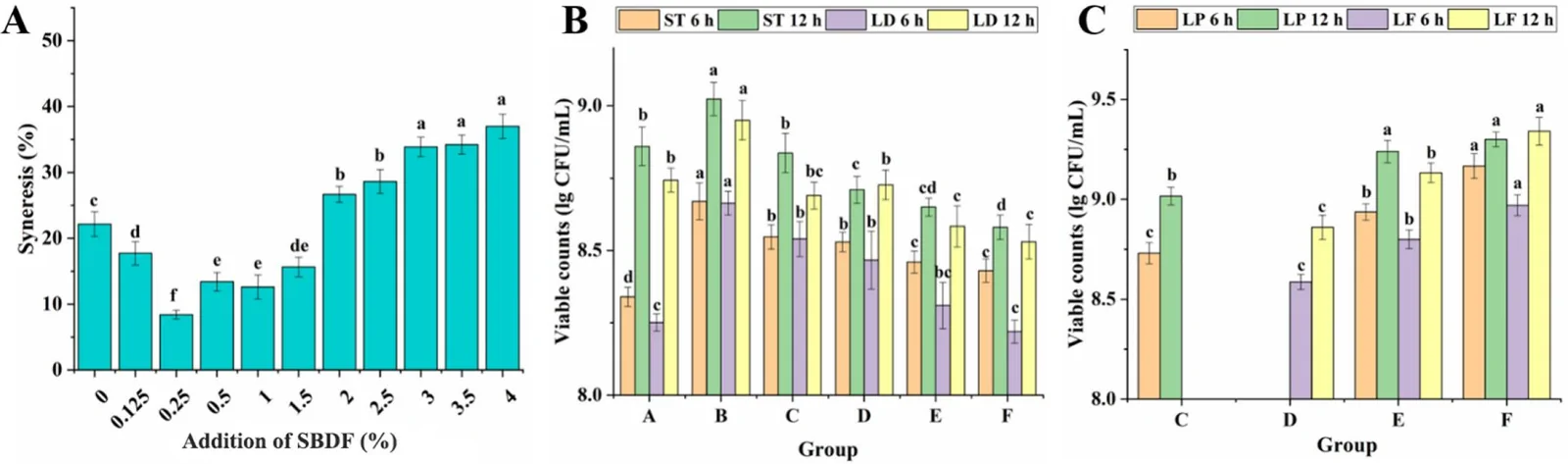
Effect on yogurt syneresis and viable bacteria count. (A): Syneresis rates at different SBDF concentrations. (B): Effect on *S. thermophilus* and *L. bulgaricus.* (C): Effect on *L. plantarum* CGMCC 1.5953 and *L. fermentum* CGMCC 1.7434. Where different lower case letters “a, b, c” indicate significant differences between groups *P* < 0.05.

### 3.2 Effects of ultrasound, binary probiotic fermentation, and SBDF on viable bacterial counts, pH, and TA

Insoluble dietary fiber in dietary fiber affects the functionality and sensory experience of food-containing products, such as dull color, low solubility and roughness. Its relatively poor bioactivity and fermentation properties also limit its application in food [40]. Therefore, the use of ultrasound to assist the fermentation of LAB may be an effective way to improve their performance in yogurt applications. As shown in Fig. 2-B, the number of viable *S. thermophilus* and *L. bulgaricus* was significantly increased in the SBDF-added yogurt (group B) as compared to the blank control (group A). In an *in vitro* analysis of longevity dietary patterns, Shi et al. found that dietary fiber had the effect of promoting the proliferation of LAB and *Bifidobacterium* [41]. However, *S. thermophilus* and *L. bulgaricus* viable counts were significantly lower in groups C, D, E, and F with the addition of probiotic as compared to group B. This may be related to competitive utilization of some nutrients by the probiotic and rapid acidification [42].

There was a significant (*P* < 0.05) increase in the number of live *L. plantarum* CGMCC 1.5953 and *L. fermentum* CGMCC 1.7434 bacteria in yogurt supplemented with binary probiotic group E as compared to single probiotic groups C and D. Significant (*P* < 0.05) increase in growth rate of *L. plantarum* CGMCC 1.5953 and *L. fermentum* CGMCC 1.7434 was observed in ultrasonically treated group F as compared to group E. The underlying mechanism of ultrasound-enhanced fermentation can be attributed to acoustic cavitation. This phenomenon generates transient microstreaming and shear forces within the fermentation medium. These physical forces create temporary pores in bacterial cell membranes, a process known as sonoporation. The increased membrane permeability facilitates nutrient uptake and metabolic waste excretion [43]. In our system, ultrasound at 100 W for 30 min likely enhanced the permeability of both starter and probiotic cells. This acceleration promoted the transport of lactose and SBDF-derived oligosaccharides. Ultrasound treatment also physically modifies the structure of SBDF by reducing particle size and increasing the exposure of soluble fiber fractions. These changes improve the prebiotic accessibility of SBDF to lactic acid bacteria [44]. The dual effect of ultrasound on microbial metabolism and substrate structure contributes to the superior fermentation performance observed in group F. As shown in Fig. 3-A, the addition of SBDF increased the pH of the yogurt compared to group A. However, this increase was counteracted by the addition of binary probiotics in group E. After ultrasound-assisted fermentation, the pH decreased even further. The total acidity of yogurt maintained a consistent trend with it (Fig. 3-B). Further supporting this, Peng et al. observed that *L. casei* Zhang promotes *Bifidobacterium lactis* V9 to generate higher levels of short-chain fatty acids in yogurt co-fermentation [17]. Similarly, Shori et al. reported a significant increase (0.18 %, *P* < 0.05) in acid production when *L. rhamnosus* and *L. plantarum* were used to ferment cashew milk yogurt [44]. In our system, *L. plantarum* CGMCC 1.5953 and *L. fermentum* CGMCC 1.7434 may similarly stimulate each other via cross‑feeding of metabolites (e.g., amino acids or exopolysaccharides), accelerating carbohydrate catabolism and acid production. Notably, ultrasonication significantly increased the conversion of free phenolic acids derived from pineapple peel dietary fiber during fermentation, particularly increasing the accumulation of ferulic and caffeic acids [45].

**Fig. 3:**
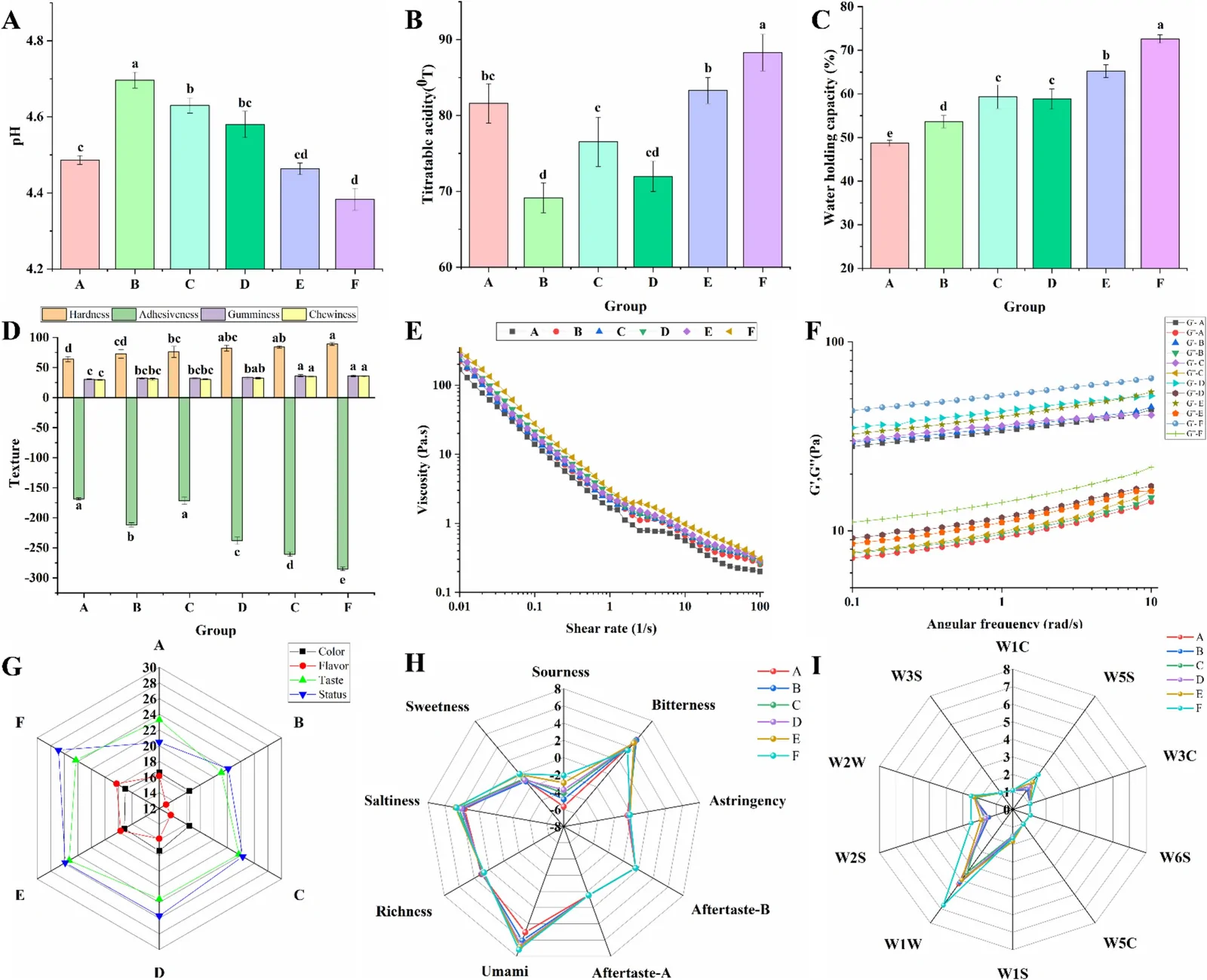
Effect of ultrasound, binary probiotics and SBDF on yogurt quality. (A): pH. (B): Titratable acidity. (C): Water holding capacity. (D): Texture. (E): Apparent viscosity. (F): Storage modulus. (G): Sensory evaluation. (H): Electronic tongue. (I): Electronic nose. Where different lower case letters “a, b, c” indicate significant differences between groups *P* < 0.05.

### 3.3 Effects on water holding capacity and texture of yogurt

Water-holding capacity is an important indicator for assessing the structural stability of fermented milk protein gels, while texture directly affects consumer acceptance [23]. The addition of SBDF significantly increased the firmness of yogurt as compared to group A (Fig. 3-C). This may be attributed to the interaction of SBDF with casein molecules in milk during yogurt fermentation, which produces gels and promotes casein interaggregation [46]. In parallel, insoluble SBDF fibers act as physical fillers that occupy interstitial spaces and immobilize water, further enhancing gel strength. The cavitation effect of ultrasound leads to a change in the structure of casein micelles, which improves the acid-gelation behavior of casein micelles and increases their viscosity and cohesion, thus improving their hysteresis [47]. Notably, group F exhibited the highest firmness and WHC, indicating a synergistic effect:ultrasound not only modifies casein structure but also promotes the release of exopolysaccharides from binary probiotics, which act as natural texturizers [48]. This result is further supported by changes in the textural characteristics of yogurt (Fig. 3-D).

### 3.4 Rheological properties

As shown in Fig. 3-E, all six yogurt formulations exhibited shear-thinning behavior, characterized by a progressive decline in apparent viscosity with increasing shear rate. This happens because higher shear forces cause protein molecules to align in the direction of flow. As a result, the interactions between molecules become weaker and ultimately lead to bond rupture and protein dissociation [23]. The incorporation of SBDF markedly enhanced the apparent viscosity of yogurt, demonstrating its effectiveness as a viscosity modifier. This improvement can be explained by the formation of hyperaggregates between SBDF particles and yogurt protein networks. However, these supramolecular structures disintegrated into smaller aggregates under high shear conditions, leading to viscosity reduction [39]. Structural analysis revealed two distinct roles for the fibres in SBDF. The coarse fibres form a three-dimensional scaffold which helps support the yoghurt matrix and maintain viscosity during storage. The fine fibres act as space fillers and help stabilise the casein network [49]. These observations align with previous reports on apple pomace-derived soluble dietary fiber, which has well-documented water-binding and viscosity-enhancing properties [24]. Notably, the ultrasound-treated binary probiotic fermentation system achieved the highest viscosity among all samples. Three synergistic factors explain this superior performance. First, the probiotic consortium produced more acid. Second, the highly methoxylated pectin in SBDF formed gels under acidic conditions. Third, ultrasound treatment induced structural changes that enhanced these effects [17].

As shown in Fig. 3-F, the storage modulus (G’) consistently exceeded the loss modulus (G”) across all yogurt formulations, confirming their dominant elastic behavior and well-developed gel structure. This viscoelastic response is characteristic of colloidal systems, where elastic properties prevail over viscous dissipation. The addition of SBDF, combined with ultrasound-assisted binary probiotic fermentation, further enhanced the elastic modulus (G’), indicating a more robust and structured gel network. The improved elasticity can be attributed to multiple synergistic mechanisms:

Protein-polysaccharide interactions played a role. The polysaccharides in SBDF formed food-grade Pickering emulsions by binding with milk proteins, reinforcing the gel matrix [44]. In addition, ultrasound treatment contributed to the effect. Both high- and low-frequency sonication promoted particle homogenization and stimulated LAB to secrete extracellular polysaccharides, which significantly enhanced the yogurt’s rheological properties [43]. Furthermore, enhanced acidification. The binary probiotic fermentation accelerated the drop in pH. This promoted the formation of a casein network. At the same time, the hydrocolloid properties of SBDF helped stabilise the resulting gel.

### 3.5 Sensory evaluation

The relationship between yogurt's structure/composition and its sensory properties remains a key research focus, particularly regarding how microstructure affects flavor compound release into the headspace [4]. Group F yogurts achieved significantly higher scores for both odor and texture. These findings align with rheological measurements, confirming that ultrasound-assisted binary probiotic fermentation of SBDF enhances yogurt texture. The improved odor profile likely results from two factors. SBDF retains characteristic sugarcane aromas during complex fermentation. Meanwhile, accelerated probiotic growth increases the metabolism of lactose, lipids, and proteins. This leads to higher concentrations of key flavour compounds, such as acetone, acetaldehyde, diacetyl, and acetic acid [31]. Color scores showed no significant intergroup variation, groups E and F demonstrated superior taste performance. These samples exhibited an optimal balance of sweetness and acidity, coupled with enhanced palatability.

### 3.6 Electronic nose and tongue

An electronic tongue and an electronic nose were used to further characterise the sensory quality of the yoghurt. Their objective was to assess the effect of SBDF addition and ultrasound-assisted binary probiotic fermentation. As shown in Fig. 3-H, there was a significant increase in sourness, umami, and sweetness and a significant decrease in bitterness in group F yogurt. However, they were not significantly different in sourness, saltiness, richness, astringency, aftertastes-A, and aftertastes-B. This is consistent with the acidity results and sensory evaluation. Several synergistic mechanisms likely contributed to this improved taste profile. This may be because the ultrasound-assisted binary probiotic fermentation broke down the macromolecular polysaccharides from the added SBDF. This breakdown produced oligofructose, low molecular weight sugars such as glucose and L-fucose, which enhance sweetness, while the metabolic activity of lactic acid bacteria generated organic acids and L-type amino acids responsible for the enhanced sourness and umami that contribute to a fresh flavour [3]. The reduction in bitterness may be attributed to the metabolic conversion of bitter peptides by the probiotic strains or their masking by the newly formed flavour compounds.

Based on functions that mimic the human olfactory system, electronic noses can recognise and distinguish subtle changes in complex odor scents [32]. The response values of W5S (sensitive to nitrogen oxides), W2W (sensitive to aromatic constituents and organic sulphides), W2S (sensitive to alcohols, aldehydes and ketones) and W1W (sensitive to inorganic sulphides) sensors were significantly higher in yogurt of group F as compared to other groups. It was shown that the content of aromatic compounds such as nitrogen oxides, sulphides, alcohols, aldehydes and ketones in yogurt increased due to the addition of SBDF and ultrasound-assisted binary probiotic fermentation. Of these, nitrogen oxides, mainly from the Maillard reaction or milk protein degradation, are known to give yogurt a nutty, roasted or malty flavor [36]. Sulphides, on the other hand, are mainly derived from the microbial metabolism of sulphur-containing amino acids (e.g. methionine, cysteine) in milk, and at low concentrations, they give a creamy corn flavor and a fruity-vegetable aroma [3]. In addition, alcohols are mainly derived from the metabolism of sugars or amino acids by LAB, and may give a floral, fruity, sweet or fermented wine aroma [13]. Furthermore, aldehydes are mainly derived from lipid oxidation and sugar degradation and are associated with apple, almond or caramel flavors [50]. Ketones, meanwhile, are derived from lactobacilli citric acid metabolism or fat β-oxidation, giving creamy and blue cheese flavors [37]. The concurrent increase in these volatile compounds, each contributing distinct desirable notes, may account for the high odor scores during sensory evaluation. Collectively, these electronic nose and electronic tongue findings provide a sensory‑based rationale for the subsequent in‑depth volatile and non‑volatile flavor compound analyses.

### 3.7 The microstructure of yogurt

Microstructural analysis offers valuable insights into yogurt gel formation, helping explain the mechanisms behind its physicochemical, rheological and textural properties [47]. The blank control yogurt (group A) showed a dense protein network with low connectivity, featuring wide pores and a rough surface (Fig. 4-A), aligning with previous reports [51]. This porous, poorly connected structure explains its lower water-holding capacity and reduced firmness observed in earlier analyses. Incorporating SBDF into yogurt produced a smoother surface and more compact, homogeneous protein clusters with reduced porosity (Fig. 4-B). Probiotic addition further decreased both the number and size of pores in the protein network (Fig. 4-C, D, E). This progressive pore reduction is primarily attributed to extracellular polysaccharides produced by the probiotics, which act as natural fillers and cross-linking agents within the protein matrix. These structural improvements in the protein network likely result from probiotic-produced extracellular polysaccharides, supporting our findings [52]. The binary probiotic groups (E and F) exhibited more pronounced pore reduction than single-strain groups (C and D), indicating that the two strains act synergistically in promoting EPS production and protein network stabilization. Furthermore, the fine soluble fiber fractions of SBDF contribute to pore filling and network compactness, while the insoluble fibers provide mechanical support. Notably, yogurt fermented through ultrasound-assisted cofermentation of binary probiotics with SBDF developed an exceptionally dense protein network containing narrow, uniformly distributed pores. This modified structure demonstrates the synergistic effects of combined processing techniques. The void regions around casein aggregates have the potential to be filled with fine SBDF, hindering whey separation and reducing coagulation [24]. This physical blocking of whey channels directly contributes to the reduced syneresis rates observed in SBDF-containing samples. In addition, SBDF not only interacts electrostatically with anionic hydrocolloids in casein micelles, but also contains soluble fibers with a porous structure that can compact casein aggregates by absorbing separated whey [5]. Furthermore, ultrasonic cavitation effects and fermentation of SBDF by binary probiotics may enhance the ability of LAB to release extracellular polysaccharides and increase the concentration of SBDF embedded in the protein network [17]. This dual action that increased EPS production coupled with greater SBDF incorporation further strengthens the gel matrix beyond what either treatment alone could achieve. Therefore, ultrasound-assisted fermentation of SBDF-containing yogurt by binary probiotics represents a highly effective strategy for controlling whey separation and enhancing protein network structure. These findings validate the approach of combining physical, biological and processing technologies to achieve superior yogurt texture and stability.

**Fig. 4:**
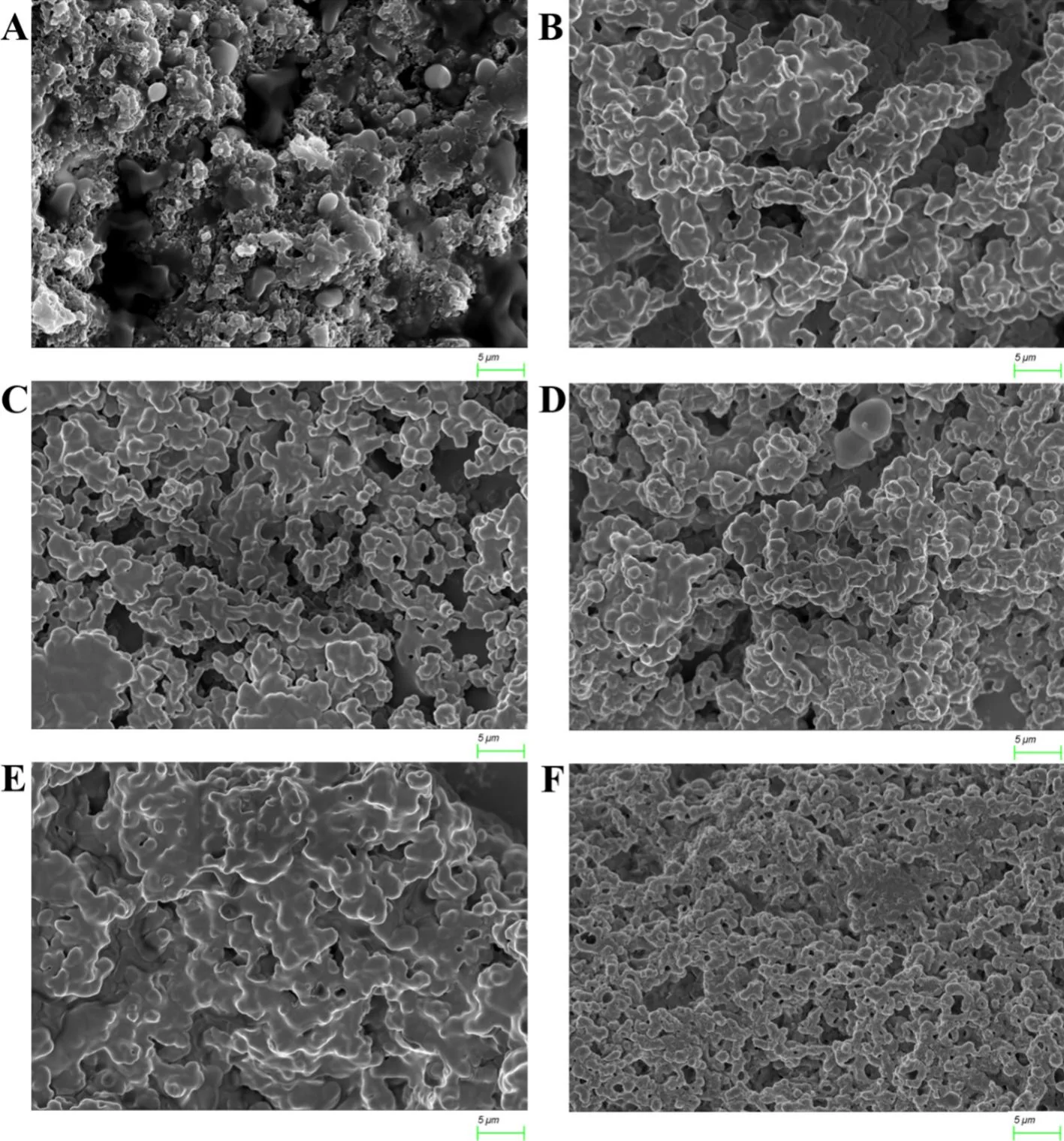
SEM micrographs (10,000 × ) of yogurt microstructure. (A): Contains only starter. (B): Add SBDF. (C): Add SBDF and *L. plantarum* CGMCC 1.5953. (D): Add SBDF and *L. fermentum* CGMCC 1.7434. (E): Add SBDF, *L. plantarum* CGMCC 1.5953, and *L. fermentum* CGMCC 1.7434. (F): Add SBDF, *L. plantarum* CGMCC 1.5953, *L. fermentum* CGMCC 1.7434, and using ultrasonic treatment.

### 3.8 Effect on volatile flavor compounds

The addition of ultrasound-assisted binary probiotic fermentation of SBDF yogurt had a significant effect on flavor, as described in 3.5, 3.6. Therefore, HS-SPME-GC–MS was used to further characterize the volatilizable volatile flavor substances in the yogurt. As shown in Fig. 5-A, the six groups of yogurt were separated along PCA1 (43.44 %) and PCA2 (24.91 %), with closer distances between the same groups indicating better inter-group reproducibility, while larger distances between the different groups suggesting differences in volatilizable flavor compounds between them. The clear separation of group F from other samples along both principal components indicated that its volatile profile had been uniquely modified. A total of 84 compounds were detected in yogurt, including alcohols, aldehydes, acids, esters, ketones, carbonyl compounds, and sulphides (Fig. 5-B). The results of trend analysis showed that Cluster 1 (ethyl acetate, trichloroethylene and benzene) and Cluster 6 (hexanoic acid and 2-nonanone) were significantly highly expressed in yogurt of group F (Table 5-C). Ethyl acetate exhibits a clean, slightly fruity wine-like aroma; trichloroethylene has a faintly sweet odor; benzene possesses a characteristic aromatic note; hexanoic acid contributes a cheesy aroma; and 2-nonanone imparts fruity, slightly greasy, cheesy, and herbal nuances [17], [37]. In addition, decane (fat-like odor), dimethyl ether (slightly sweet odor characteristic of ethers), and 4-methyl-2-hexanol (sweet or fruity aroma) were also significantly up-regulated and VIP > 1 compared to the other groups. This could be the reason for the high sensory odor scores of yogurt in group F.

**Fig. 5:**
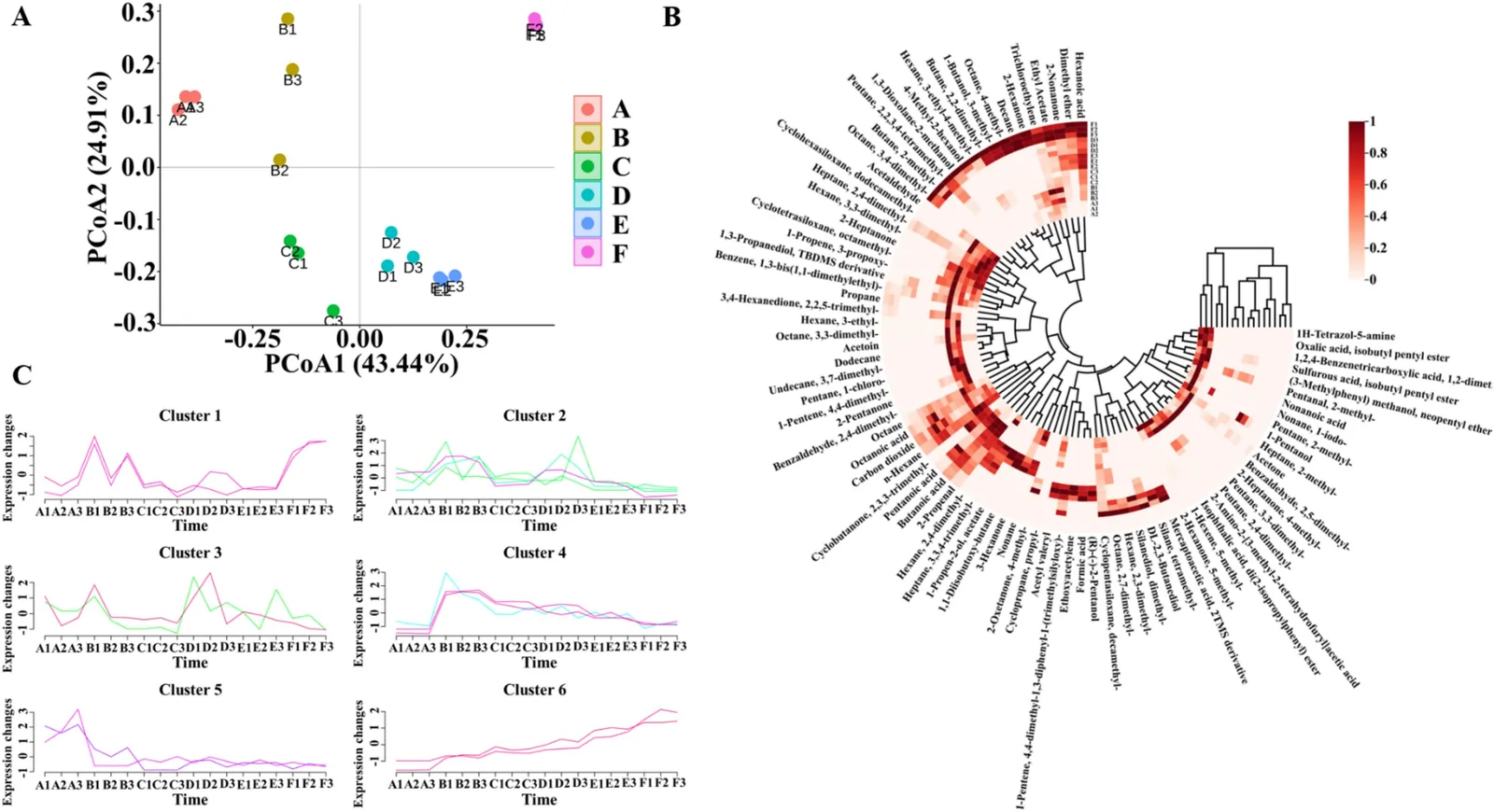
Volatile flavor compounds. (A): PCoA analysis. (B): Heatmap. (C): Trend analysis.

The enrichment of esters, acids, and ketones in group F resulted from ultrasound‑assisted binary probiotic fermentation. Ultrasound boosts ester‑synthesizing enzyme activity in LAB, while the binary probiotics supply complementary pathways: *L. plantarum* forms esters, and *L. fermentum* produces short‑chain fatty acids. Their co‑cultivation with ultrasound shifts volatiles toward fruity and cheesy notes, aligning with the elevated W2S/W2W responses and odor scores. This confirms that ultrasound‑assisted binary probiotic fermentation with SBDF enhances key odor-active compound production, explaining the superior sensory performance of group F yogurt.

### 3.9 Effects on non-volatile flavor compounds

Based on these results, ultrasound-assisted binary probiotic fermentation of SBDF-fortified yogurt significantly influenced yogurt quality. To further elucidate the underlying mechanisms and identify key flavor compounds, UHPLC-MS combined with untargeted metabolomics was performed. VIP ≥ 1, p-value < 0.05, and fold change ≥ 1.5 or ≤ 0.667 were selected as criteria for screening biomarkers according to Wang et al. [22] and Peng et al. [17]. As shown in Fig. 6-A, the six yogurts differed considerably in metabolites, with large distances between the different groups.

**Fig. 6:**
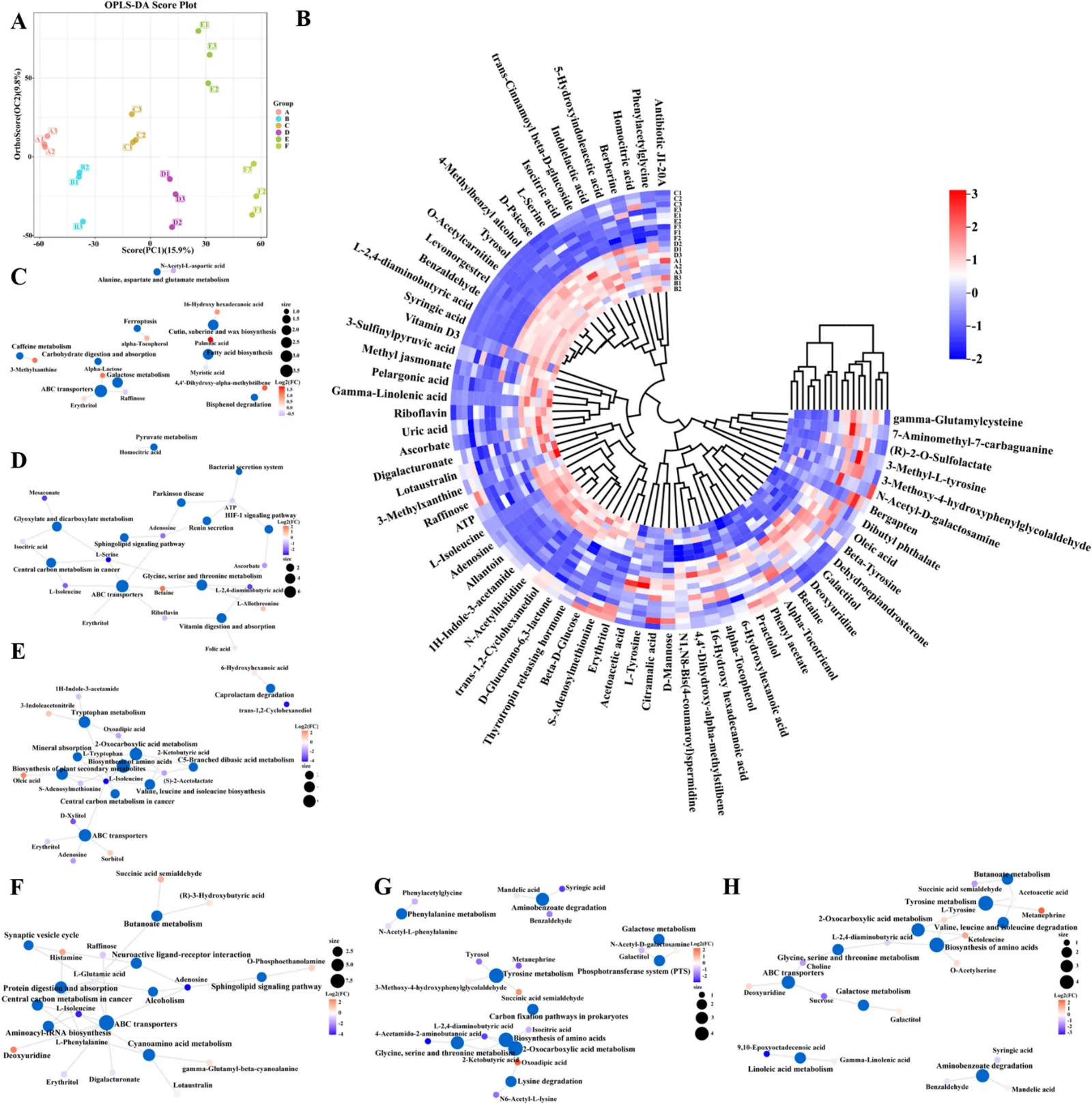
Non-volatile flavoring compounds. (A): OPLS-DA analysis. (B) Heatmap. (C): Differential pathway network diagram.

As shown in Fig. 6-B, there are a total of 30 differential metabolites between group A and group B. Among them, N1,N8-bis(4-coumaroyl)spermidine (BBC), β-N-acetylglucosamine, L-erythrulose, isovaleric acid, palmitic acid, α-lactose, and erythritol upregulated by 246.91, 4.72, 4.42, 4.25, 3.4, 2.35, 1.85 times. BBC can slow down the aging process by inducing autophagy, an intracellular “regeneration” process that helps to remove damaged or aged cellular components, thereby extending life span. In addition, studies have shown that dietary BBC can cross the blood–brain barrier and increase mitochondrial respiration in neuronal tissue, improving cognitive function, memory and discrimination [53]. This may be the main reason for the increase in the number of live bacteria. In addition, β-N-Acetylglucosamine has been shown to increase skin hyaluronic acid content, reduce joint pain, improve symptoms of diabetes and hepatitis, and boost immunity [54]. Isovaleric acid is also commonly used in the formulation of food flavorings to enhance flavor in certain beverages, confectionery and pastries [8]. Notably, L-erythrulose, α-lactose, and erythritol may be the main components of SBDF. These SBDF-derived compounds contribute to mouthfeel and sweetness, supporting the enhanced taste acceptability of group F yogurt. This is mainly regulated by cutin, suberine and wax biosynthesis, galactose metabolism, ABC transporters, fatty acid biosynthesis, caffeine metabolism pathways (Fig. 6-C).

Compared with group B, a total of 54 differential metabolites were found in group C. Among them, betaine (4.04-fold up-regulation) under adverse conditions such as low water, high salinity or extreme temperature, betaine is able to stabilize the structure and function of biomolecules, preventing cell degeneration and functional inactivation. In addition, phenyl acetate (1.88-fold upward adjustment) is widely used as a synthetic flavoring with an aroma similar to that of a rose with a strawberry-like sweetness. L-Allothreonine (up 1.85 times) is necessary to maintain nitrogen balance in the body and promote normal growth and development. This is mainly influenced by Glycine, serine and threonine metabolism and ABC transporter pathway (Fig. 6-D). Galactitol, a prebiotic that promotes the growth of LAB [42], was up-regulated 12.95-fold in group D compared to group B. It is mainly regulated by the biosynthesis of amino acids, valine, leucine and isoleucine, ABC transporters, and galactose metabolism (Fig. 6-E).

Galactitol was upgraded 11.91-fold, while L-phenylalanine, L-glutamic acid, L-allothreonine, (S)-beta-tyrosine, L-theanine, and L-isoleucine were downgraded 1.73, 2.13, 3.03, 4.25, and 14.28-fold, respectively. Notably, the extent of galactitol upregulation varied among probiotic treatments. Group D showed a 12.95-fold increase, whereas group F showed an 11.91-fold increase. This suggests that *L. fermentum* may be particularly efficient in galactitol biosynthesis, while the presence of *L. plantarum* in binary groups may shift metabolic flux toward other complementary pathways. The type of dietary fiber also influences these metabolic outcomes, as SBDF-derived oligosaccharides serve as substrates for galactitol production through the galactose metabolism pathway [55]. In addition, due to its slow absorption into the body, galactitol has a low impact on blood sugar levels and is also suitable for people with diabetes. Its good moisturising properties can prevent food from drying out and hardening too quickly, extending its shelf life. Galactitol is known to increase the viscosity of solutions and stabilize food structures by acting as a thickening agent. The significant upregulation of this polyol in group F is therefore likely to contribute to the enhanced texture and sweetness perception observed in this group, although a direct causal link remains to be firmly established. The significant upregulation of sweet‑tasting polyols directly explains the increased sweetness perception in group F. Certain amino acids (L-glutamic acid, L-theanine, and L-isoleucine) have been reported to be able to promote bacterial growth and proliferation, and can help bacteria to construct proteins with correct structure and function [16]. This further supports the ability of binary probiotics to promote growth when fermenting SBDF yogurt. The marked decrease in bitter amino acids and their derivative correlates well with the reduced bitterness signal detected by the electronic tongue. Its summary metabolic pathway in this system is shown in Fig. 7. Meanwhile, oxoadipic acid, pentadecanoic acid, and 2-ketobutyric acid were up-regulated by a factor of 4.95, 4.49, and 1.86, respectively, which could be the main reason for the decrease in pH and acidity. The accumulation of these organic acids contributes to the increased sourness perception, while the downregulation of umami‑related amino acids appears contradictory to the increased umami scores; however, the overall taste profile is likely modulated by the synergistic effects of multiple metabolites, including sweeteners and the specific organic acid profile. Ultrasound-assisted fermentation further increased the 2,6-dimethoxyphenol (woody medicinal odor, smoky phenolic aroma) and galactitol content by 7.25 and 1.81 times. This is mainly related to tyrosine metabolism, glycine, serine and threonine metabolism, amino acid biosynthesis, galactose metabolism and lysine degradation metabolic pathways (Fig. 6-F, G, H). These integrated metabolomic and electronic tongue analyses demonstrate that ultrasound‑assisted binary probiotic fermentation of SBDF‑fortified yogurt reshapes the non‑volatile metabolome, leading to improved taste characteristics as captured by both instrumental and sensory evaluations.

**Fig. 7:**
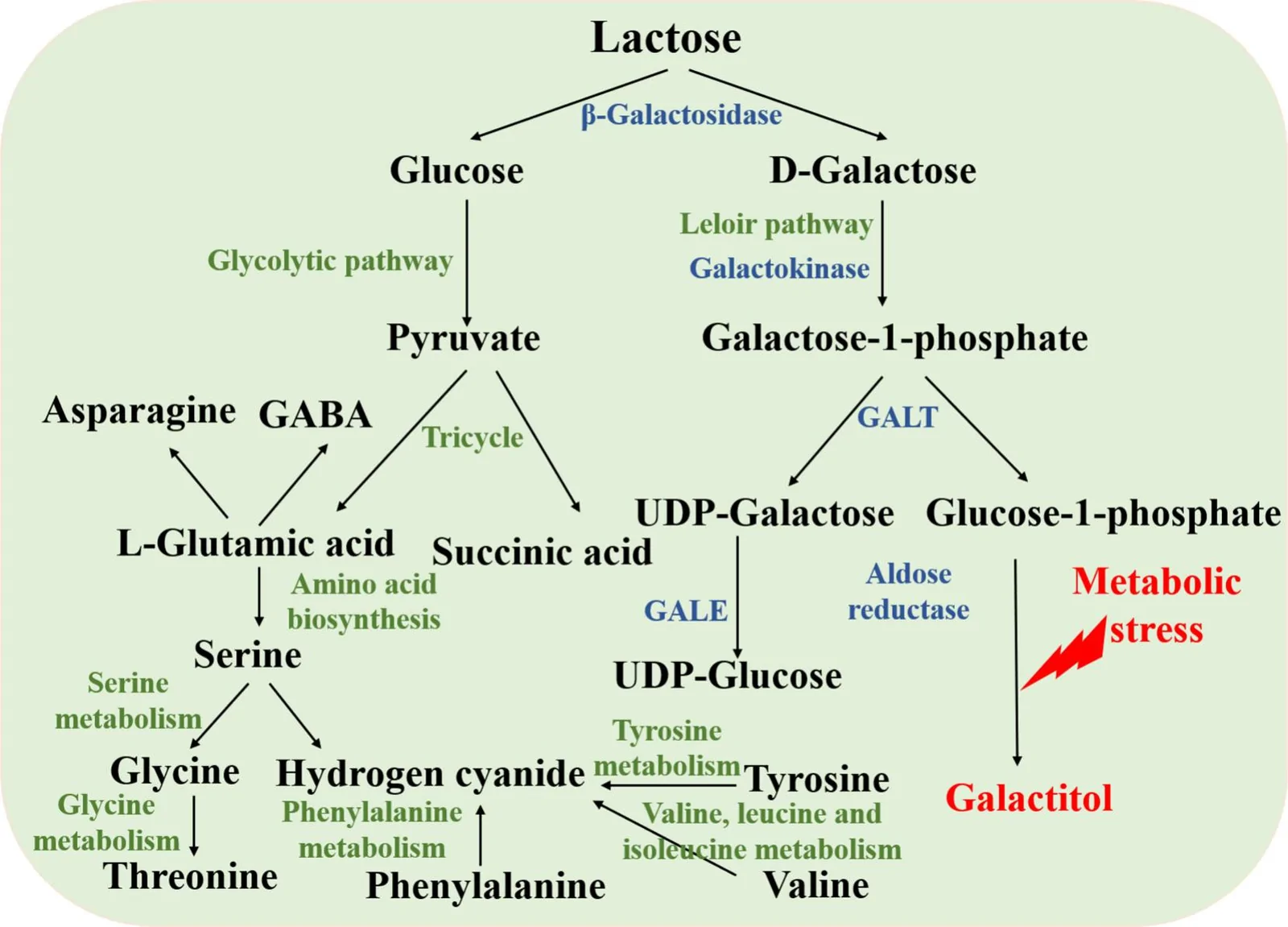
Galactitol and the key amino acid metabolic pathways in ultrasound-assisted binary probiotics fermentation of synbiotic yogurt.

### 3.10 Shelf-life stability of yogurts during refrigerated storage

To evaluate the storage stability of the six yogurt formulations, pH, titratable acidity (TA), and total viable counts were monitored over 28 days at 4 °C. As shown in Fig. 8-A, the pH of all yogurts decreased progressively during storage, with group B consistently maintaining the highest pH throughout storage (from 4.64 to 4.22) due to the buffering effect of SBDF addition. Notably, group F exhibited the most stable pH profile. Correspondingly, as shown in Fig. 8-B, the TA of group F increased most slowly (from 72.5 to 85.2°T, an increase of only 12.7°T), while groups D and E showed the largest increases. These results indicate that ultrasound-assisted binary probiotic fermentation effectively mitigated post-acidification during cold storage. Regarding total viable counts (Fig. 8-C), groups E and F, containing all four strains, exhibited the highest initial counts at day 0, reaching 10.14 and 10.22 lg CFU/mL, respectively. Among all groups, group F demonstrated the most stable viability during storage, decreasing by only 3.61 lg units over 28 days (from 10.22 to 6.61 lg CFU/mL) and maintaining the highest viable count at day 28, exceeding the Chinese national standard requirement of ≥ 6.0 lg CFU/mL (GB 19302–2010). In contrast, group B fell below the standard at day 28 (5.82 lg CFU/mL). The shelf-life data further corroborate the conclusion that ultrasound-assisted binary probiotic fermentation with SBDF contributes to enhanced yogurt stability during cold storage, as reflected by the mitigated post-acidification and improved probiotic survival in group F. These findings reinforce the potential of this synergistic strategy for developing synbiotic yogurt with extended shelf-life quality.

**Fig. 8:**
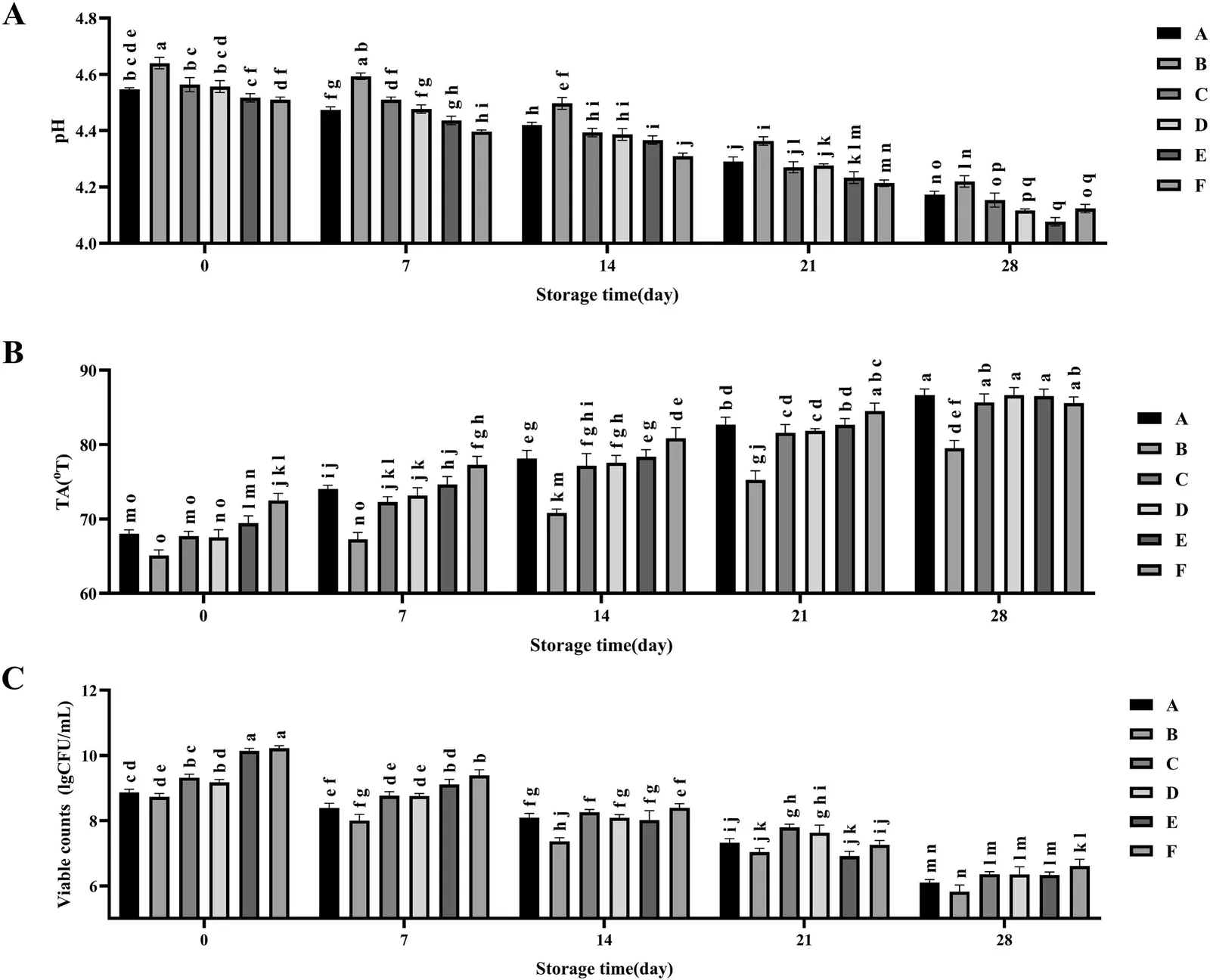
Shelf-life stability of six yogurt formulations (groups A–F) during 28 days of refrigerated storage at 4 °C. (A): pH. (B): Titratable acidity (°T). (C): Total viable counts (lg CFU/mL). Values are expressed as mean ± SD (n=3). Different lowercase letters indicate significant differences between groups at the same time point (*P* < 0.05).

## 4 Conclusion

In this study, the effect of ultrasound-assisted binary probiotic fermentation on the quality of SBDF-added yogurt was investigated. While the conventional extraction method used herein serves the purpose of generating a stable SBDF reference material for mechanistic inquiries, our findings also lay the groundwork for future optimization of green extraction technologies and their industrial application. The results demonstrated that adding 0.25 % (w/v) SBDF significantly reduced yogurt syneresis. Furthermore, ultrasound-assisted fermentation with *L. plantarum* CGMCC 1.5953 and *L. fermentum* CGMCC 1.7434 improved the yogurt’s texture, rheological properties, microstructure, and sensory evaluation scores. This combination promoted a more compact and uniform protein network, as visualized by microstructure analysis, by stimulating exopolysaccharide production and enhancing the integration of SBDF-derived compounds. Mechanistically, the improved texture and mouthfeel are likely associated with the significant upregulation of galactitol, a sweet polyol known to enhance viscosity and stabilize food structure. Other SBDF-derived compounds, such as L-erythrulose and erythritol, may also contribute. However, further validation through exogenous supplementation assays is warranted to confirm the causal role of galactitol, as the present study relies on correlative metabolomic data. Concomitantly, the enhanced flavor profile resulted from the metabolic conversion of key amino acids, as evidenced by the decrease in L-phenylalanine, L-glutamic acid, and L-allothreonine. This drove the accumulation of desirable volatile compounds such as ethyl acetate, hexanoic acid, and 2-nonanone. These integrated physicochemical and biochemical improvements were primarily orchestrated through the modulation of key metabolic pathways, including galactose metabolism for structure and sweetness, and amino acid metabolism (tyrosine, glycine-serine-threonine, lysine degradation) for flavor development. This study not only provides a mechanistic framework for producing a premium-quality synbiotic yogurt but also demonstrates a viable strategy for the high-value utilization of sugarcane bagasse dietary fiber.

## Declaration of generative AI in scientific writing

Generative AI and AI-assisted technologies should only be used in the writing process to improve the readability and language of the manuscript.

## CRediT authorship contribution statement

**Hui Zhou:** Writing – original draft, Methodology, Data curation. **Wenying Yuan:** Writing – review & editing. **Chengfeng Xu:** Investigation. **Xiefei Li:** Visualization, Supervision, Resources. **Zhen Wu:** Data curation. **Daodong Pan:** Writing – review & editing, Conceptualization. **Jie Luo:** Project administration, Funding acquisition, Conceptualization. **Xiankang Fan:** .

## Declaration of competing interest

The authors declare that they have no known competing financial interests or personal relationships that could have appeared to influence the work reported in this paper.
